# Treatment of Dental Pulp

**Published:** 1863-10

**Authors:** Geo. B Snow


### TREATMENT OF DENTAL PULP.

BY GEO. B. SNOW.

Read before the Western N. Y. Dental Association.

*Mr. President and Gentlemen of the Dental Association of Western New York.*

Having been appointed to read an essay before you, I propose to take for my subject the treatment of the Dental Pulp.

We come daily, almost hourly, upon cases of disease of this delicate and sensitive organism. Sometimes, indeed too often, the only treatment they receive is what may be obtained from a pair of forceps and a good strong pull. *That* tooth is forever put beyond the possibility of aching again; but it may be that the patient loses a tooth whose services, either in speech or mastication, he will find has been of inestimable value, and whose place can not be fully restored.

With regard to the possibility of saving teeth, in which the pulp is exposed, diseased or destroyed, the result achieved by those who have given the subject proper attention, warrant me in saying, that with proper care in the performance of the operations required, certainly nine out of ten may be rendered useful and comfortable for years. A great deal may be done to ensure success, by exercising proper care in the selection of cases. The tooth should have an antagonist, so that it may be used in mastication. The gums and surrounding parts should be healthy, and the general health of the patient should be good. Those who are predisposed to active inflammatory disorders, or who appear peculiarly liable to dental exostosis, or who have a syphilitic taint, are bad subjects for the treatment under discussion.

The first step in the treatment of the dental pulp is an accurate diagnosis. In most cases this is an easy matter, but in others it is quite difficult. If an actual exposure of the pulp is visible, or can be made so by the excavator, we can easily arrive at a definite conclusion in the matter; but we are often here as in legal cases, obliged to rely upon circumstantial evidence. If the patient has suffered pain from the tooth, a great deal may be learned from its character. If continued pain results from packing cotton tightly into the cavity, the pulp is probably exposed. Another symptom is pain induced by throwing a stream of lukewarm water into the cavity with a syringe.

With regard to the treatment necessary in case the pulp is at all diseased, or even fully exposed, the best plan is agreed by all to be its destruction and removal from the tooth. But when it is in a healthy condition, and either very slightly exposed or protected by a thin lamina of bone, attempts are sometimes made to preserve it alive, by capping it with horn, sheet lead, gutta percha or asbestos, or by "bridging" the filling as it is called, so that it will not touch the bottom of the cavity in the vicinity of the point of exposure. From some reason, however, the failures appear to be quite as numerous as the successes in the methods of treatment last mentioned, although some eminent dentists appear to have considerable faith in them.

If we find a tooth in which we have reason to believe the pulp nearly exposed, but which has never given any pain, and which is not sensitive to percussion, I think it best to excavate the bottom of the cavity but very little; leave the decay there if need be, but saturate it well with creosote and place over it some non-conducting substance. The tooth should only have a temporary filling, one that can be removed with ease if there should be any trouble, and the chances are that it may be replaced in time by a better one, and the tooth saved.

The practice of excising the live pulp, is one that does not require extended notice at this time. Other and less painful methods have almost entirely replaced it, although it may still be regarded as expedient in favorable and easily accessible cases. The common agent now used for the destruction of the dental pulp is the arsenical paste, which in experienced hands accomplishes the result, with but little pain and great certainty. This preparation is made up after different formulæ, the most usual being about 3 parts of arsenious acid, to 2 of morphia by weight, well triturated with creosote, enough to form a paste of the consistence of thick cream. Another recipe uses cobalt, a variable compound of metallic arsenic, arsenious acid, etc., instead of arsenious acid. Sometimes the morphia is omitted entirely. Before we apply the paste, we must be certain that the pulp is living and actually exposed. The exposure should be as full as practicable.

Some judgment should be exercised as to the amount of the paste applied, as no more should be used than is necessary to produce the desired effect. The usual direction is to take what will adhere to a pellet of cotton as large as a pin's head. The quantity will vary somewhat with different persons and with different teeth. The paste should be placed directly in contact with the pulp, and should be secured in such a manner that no pressure will be brought upon it. After from twenty-four to forty-eight hours have elapsed, the application may be removed from the tooth, which may now be left without further treatment for a week or ten days. In this time a separation will take place between the living and dead parts; after which, the dead pulp may almost always be removed with ease. A good instrument for this purpose can be made from a small sized broach. It should first be well annealed, and should then have a small hook turned upon its point. The barbed instruments sold for the purpose are very apt to break in the tooth, and are often too large and clumsy to be of any use. The instrument is to be passed well up into the pulp cavity, and rotated several times, so that the hook will become entangled in the membranes of the pulp. After the pulp is entirely removed, the fangs of the tooth should be filled with a cotton thread saturated with creosote, which should be covered by a filling of gutta percha. If this will remain a week or so without causing any pain or soreness, the tooth may be filled without any fear of unpleasant consequences. I would here remark, that it is best not to fill one of these "nerve cases," as they are familiarly called, if there is any pain about them, or tenderness upon striking them gently with the finger or an instrument.

We sometimes find upon examination of an aching tooth, that the pulp is in a state of suppuration. If so, it should at once be removed from the tooth, if possible, and the tooth treated with creosote until the cotton upon which it is applied has no bad smell after remaining in the tooth for two or three days, and until all pain and soreness have disappeared.

By far the greater number of cases of alveolar abscess require merely an extension of the treatment last mentioned. If there is a fistulous opening in the gum, or any appearance of a collection of pus, it should be freely opened with a lancet.

If possible, creosote should be forced through the fang until it makes its appearance at the opening in the gum. The tooth including the pulp cavity should be cleansed as thoroughly as possible. If there is a very offensive smell about it, tincture of iodine may be used as a disinfectant. The pulp cavity should now be filled with cotton saturated with creosote, and the opening in the gum should receive the same treatment, which will require renewal every two or three days.

Sometimes creosote seems to increase instead of diminish the pain and soreness in this class of cases. If so, a few small crystals of chlorate of potash passed as far up the root as possible will benefit it. This will generally alleviate the pain, even in the formative stage of alveolar abscess, if applied in this manner; but as an external application it is nearly useless.

After having restored the tooth as nearly to a state of health as possible, we proceed to fill the fangs. We first cut away the tooth sufficiently to render the canals in the fangs easily accessible, and if necessary enlarge them by means of a suitable drill. We next prepare a number of small broaches by rolling small strips of non-adhesive gold or tin foil upon their points, thus forming small cylinders, which should be small enough to pass to the extremity of the root, and large enough to prevent all danger of their passing through the foramen. The tooth is now thoroughly dried out, and one of the broaches, its point previously dipped in creosote, is passed to the extremity of the root. By turning it in the right direction, the foil upon its point is partially unwound and loosened, so that the broach may be withdrawn. A suitable plugger now follows, the foil is consolidated, and the process repeated until the fang is filled.

One important element of success in the treatment of this class of cases, is in filling the fangs at the proper time. The patient should be in his ordinary health, if possible. Exposure to extreme fatigue, or catching a severe cold at this time, or sometimes a long ride against a cold wind, either directly before or after the operation, will go a great way against success. The operation, no matter how carefully it may be performed, is productive of a kind of shock to the tooth and surrounding parts, and they should have time to recover their natural tone. So it is sometimes best, after filling the fangs, to defer filling the cavity of decay until some other time.

